# Case report: Castleman’s disease involving the renal sinus resembling renal cell carcinoma

**DOI:** 10.3389/fsurg.2022.1001350

**Published:** 2022-09-05

**Authors:** Enlong Zhang, Yuan Li, Ning Lang

**Affiliations:** ^1^ Department of Radiology, Peking University Third Hospital, Beijing, China; ^2^ Department of Radiology, Peking University International Hospital, Beijing, China

**Keywords:** hyaline vascular type, renal sinus, computed tomography urography, case report, Castleman disease

## Abstract

**Introduction:**

Castleman's disease (CD) is a rare benign lymphoproliferative disease that frequently involves the mediastinal thorax and the neck lymph nodes. It rarely affects extrathoracic presentations, with even fewer presentations in the renal sinus.

**Patient concerns:**

In this report, we present a case of a 40-year-old woman with no significant past medical history who presented Castleman's disease arising in the renal sinus.

**Diagnosis and interventions:**

The patient visited our hospital with the chief complaint of left renal sinus lesion after renal ultrasonography by regular physical examination. Subsequent abdominal computed tomography urography revealed a soft tissue mass with heterogeneous obvious enhancement in the sinus of the left kidney, which was suspected to be a renal malignant tumor. Hence, the patient underwent a left radical nephrectomy. Histological examination revealed hyperplastic lymphoid follicles in the renal sinus and was finally diagnosed as Castleman's disease of the hyaline vascular type.

**Outcomes:**

Five days after the surgery procedure, the patient was discharged.

**Conclusion:**

Due to the low incidence of Castleman's disease in renal sinus, there is a strong likelihood of missed diagnosis or misdiagnosis, and it is, therefore, important to be aware of the risk. Heightened awareness of this disease and its radiographic manifestations may prompt consideration of this diagnosis. Therefore, we explored the radiologic findings to find out some radiologic features suggesting this condition to help clinicians to schedule nephron-sparing surgery in the future.

## Introduction

Castleman's disease (CD), also known as giant lymph node hyperplasia, is a rare, and clinically and histopathologically heterogeneous group of disorders sharing in common an abnormal lymph node pathology of unknown etiology (1). The disease occurs in two clinical forms: unicentric CD (UCD) and multicentric CD (MCD), and is divided pathologically into three types: hyaline vascular, plasma cell variants, and mixed type. UCD is the most common, occurring at a rate of 16 per million person years and occurs at any age (2); approximately 75% of these cases are hyaline vascular variant and is usually curable by surgical resection. MCD is classically associated with systemic symptoms and a poorer prognosis. CD frequently involves the mediastinal thorax and the neck lymph nodes (3). It rarely affects extrathoracic presentations, with even fewer presentations in the renal sinus. Preoperative diagnosis is difficult because diagnostic imaging alone cannot distinguish CD from other diseases due to the lack of tumor-specific signs. Here, we report a case of hyaline vascular type CD with renal sinuses involved. By reviewing the literature, we hope to find out the imaging features to assist in the preoperative qualitative diagnosis of this disease and as a reference to clinicians in the future. The present case report is developed according to the CARE checklist (4, 5).

## Case presentation

**Figure 1:**
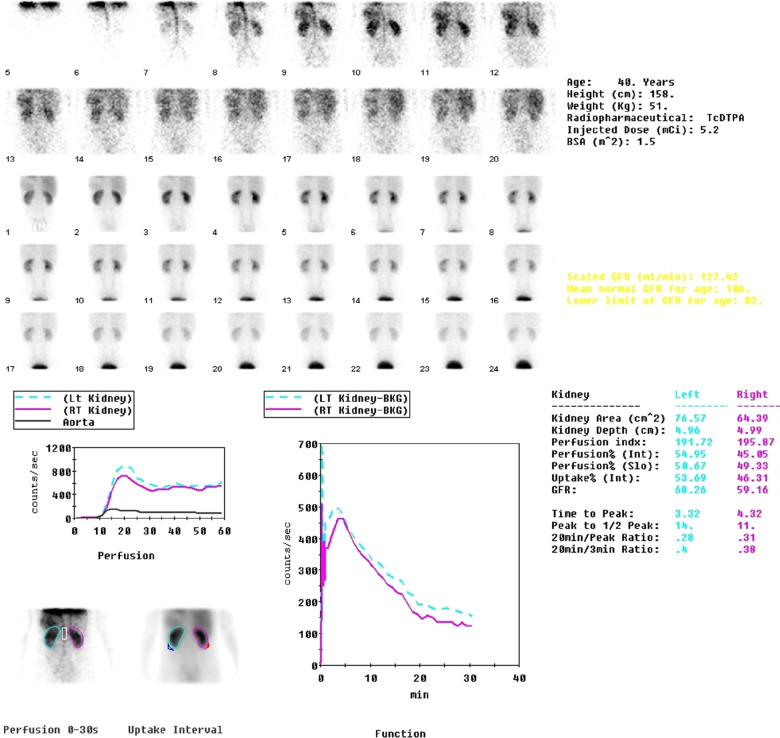
99mTc DTPA renal scintigraphy shows normal renal blood flow.

**Figure 2:**
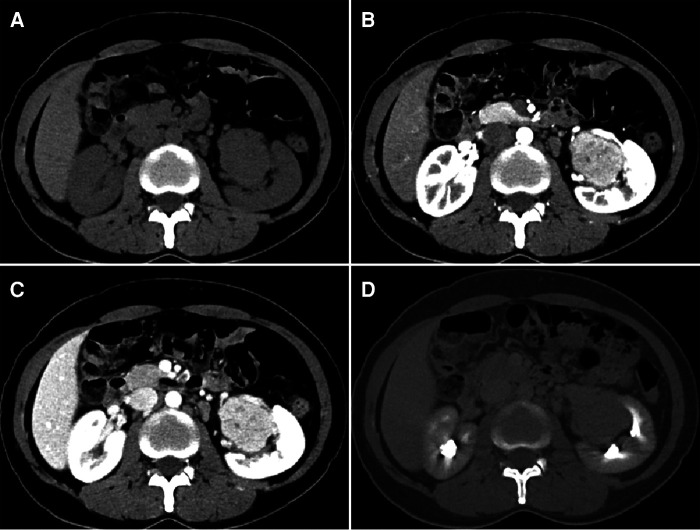
CTU reveals a soft tissue mass in the left renal sinus with heterogeneous homogeneous enhancement after injection of contrast media. (**A**) Unenhanced CT scan; (**B**) corticomedullary phase; (**C**) nephrographic phase; (**D**) excretory phase.

A 40-year-old female patient presented to our hospital with the chief complaint of left renal sinus lesion after renal ultrasonography was performed as part of a healthy physical examination. The patient had no symptoms of infection such as fever, night sweats, or other clinical symptoms. Physical examination also showed no obvious abnormalities. Laboratory examinations such as C-reactive protein (CRP), erythrocyte sedimentation rate (ESR), and interleukin-6 (IL-6) showed no significant abnormalities. Urinalysis findings were within normal limits, with urine cytology being normal, and 99mTc DTPA renal scintigraphy images showed normal renal blood flow (Figure 1). Abdominal computed tomography urography (CTU) revealed a 4.7 × 4.0 × 3.8 cm soft tissue mass in the left renal sinus with heterogeneous obvious enhancement (CT: 42HUPS, 136HUCMP, 122HUNP, 85HUEP) (Figure 2). The mass was adjacent to the renal parenchyma, and the boundary was unclear. The left renal artery and vein, renal pelvis, and calyces showed compression changes. No obvious signs of dilated hydronephrosis were observed in the urinary tract. Multiple enlarged lymph nodes were seen retroperitoneum. A small amount of effusion was observed in the pelvic cavity. A renal malignant tumor was suspected, and the patient was scheduled for a left radical nephrectomy. She did not have a family history of renal cell carcinoma or genetic abnormalities. The cut surface of the renal sinus in the middle of the kidney revealed a grayish-yellow nodular mass (about 5.2 × 4.0 × 3.5 cm) with spotty hemorrhage. The renal pelvis is unclear and the mass is adjacent to the renal parenchyma. The mucosal layers of the renal pelvis, calyces and ureter were intact, and no evidence of urothelial malignancy. Histological and immunohistochemical findings were compatible with a diagnosis of CD of the hyaline vascular type. The patient was discharged on day 5.

## Discussion and conclusion

CD is a rare disease characterized by the massive growth of the abnormal lymph node (1). The pathogenesis of CD remains unknown. The possible mechanisms include chronic low-grade inflammation, immunodeficiency state, and autoimmunity. CD can be classified clinically as UCD and MCD, and pathologically as hyaline vascular, plasma cell variants, and mixed type. UCD is the most common, occurring at the rate of 16 per million person years, occurs at any age (2), and is usually common in young individuals. The most common pathological type is hyaline vascular type, which is characterized by small and transparent vascular follicles and proliferation of capillaries between the hair follicles. Contrast-enhanced CT showed significant enhancement, even to the vascular enhancement level. The findings of UCD on 99mTc DTPA renal scintigraphy have not been well-reported. Song et al. reported a case of retroperitoneal extrarenal UCD. 99mTc DTPA renal scintigraphy shows that both kidneys are functioning normally. However, on both the posterior 2-s/frame blood flow images and the 1-min/frame functional images, UCD peaked at about the same time as the cortex of the kidneys and gradually decreased at a similar rate to the kidney activity. It seemed that the UCD exerted a mass effect and compressed the right kidney laterally (6). These findings were compatible with our case. However, the mass in our case was small, there was no obvious mass effect, and no lesions were found in the blood flow images and functional images. This type is present in 90% of patients with UCD and its most common location is the mediastinum. Usually, the diagnosis of UCD is incidental, most cases of UCD are asymptomatic, and often no laboratory abnormalities are present (7). Our patient was asymptomatic.

Surgical resection for UCD is the most appropriate first-line intervention, according to uniform panel consensus. Important considerations are the location, the resectability, and the degree to which the enlarged lymph node causes symptoms by compressing neighboring anatomy or inducing a systemic inflammatory syndrome. Recurrence of UCD after complete surgical removal is rare (7). In our case, the tumor was adjacent to the renal parenchyma, and the boundary was unclear. In addition, multiple enlarged lymph nodes were seen in the retroperitoneum. Therefore, the tumor was mistaken as a malignant lesion originating from the kidney with lymph node metastasis before the operation, so radical nephrectomy was performed. In a similar report by Radfar et al., they removed the mass laparoscopically and spared the left kidney, but analysis images showed a certain distance between the lesion and the renal parenchyma, suggesting that the tumor did not originate from the kidney, and radical nephrectomy was not performed (8). The plasma cell type is characterized by patches of plasmacytic between large follicles. Compared with the hyaline vascular type, it has less vascular density and is associated with many signs and symptoms. The less common MCD is usually found in older people, about 80%–90% of these cases are plasma cell variants. Most patients with MCD present with fever, malaise, anemia, elevated ESR, frequent abdominal lymph nodes enlargement, systemic symptoms, hepatosplenomegaly, and systemic lymphadenopathy. The mixed type is characterized histologically by a combination of hyaline vascular and plasma cell morphology.

CD rarely affects extrathoracic presentations, with even fewer presentations in the renal sinus. To the best of our knowledge, only a few cases have been reported (8–16). The clinicopathological features of the reported cases are shown in Table 1. There were 11 patients (8 males and 3 females). However, CD is generally gender-neutral. The median age of CD is 40 years old, and the mean age of sinus involvement was 62 years. The main manifestations were abdominal pain (*n* = 2), weight loss (*n* = 2), microscopic hematuria (*n* = 2), and anorexia (*n* = 1); however, five patients were asymptomatic and the clinical type was UCD. Abdominal pain can be seen both in UCD and MCD patients, weight loss almost in MCD patients, and microscopic hematuria in UCD patients. Seven cases were from a single center and four cases were multi-center. There were seven cases of plasma cell type, three cases of transparent vascular type, and one case of mixed type. The present patient, a 40-year-old female, was asymptomatic of clinically UCD and pathologically transparent vascular type. All mass sizes are less than 5 cm.

**Table 1:** Clinicopathological and imaging characteristics of previously reported cases of CD with renal sinus involvement.

References	Gender	Age (year)	Symptoms	Sinus	MRI			CT		Clinical type	Pathological type
					T1WI	T2WI	DWI	Size (cm)	Enhancement		
Nolan et al. (9)	M	62	Abdominal pain, microscopic hematuria	Unilateral	N/A	N/A	N/A	N/A	N/A	UCD	PC
Nagahama et al. (10)	M	79	Anorexia, weight loss	Left	Isointense	Hypointense	N/A	N/A	Mild	MCD	PC
Nishie et al. (11)	M (*n* = 2), F (*n* = 1)	73/70/65	Weight loss (*n* = 1), asymptomatic (*n* = 2)	Bilateral (*n* = 2), unilateral (*n* = 1)	Isointense (*n* = 2)	Hypointense (*n* = 2)	N/A	3.0–4.5	Mild	UCD (*n* = 1), MCD (*n* = 2)	PC (*n* = 2), MF (*n* = 1)
Park et al. (12)	M	50	Abdominal pain	Right	N/A			N/A	Mild	MCD	HV
Jang et al. (14)	M	64	Microscopic hematuria	Left	N/A	N/A	N/A	2.5–4	Mild	UCD	PC
Kim et al. (13)	M	59	Exertional dyspnea	Left	Isointense	Hypointense	Hyperintense	N/A	Mild	UCD	PC
Guo et al. (15)	F	62	Asymptomatic	Right	Isointense	Hypointense	Hyperintense	2.8–4.5	Mild	UCD	PC
Li et al. (16)	M	56	Asymptomatic	Right	N/A	N/A	N/A	3.5–4	N/A	UCD	HV
Radfar et al. (8)	F	32	Dull left flank pain	Left	N/A	N/A	N/A	7	N/A	N/A	N/A
Present	F	40	Asymptomatic	Left	N/A	N/A	N/A	3.8–4.7	Mild	UCD	HV

M, male; F, female; N/A, not available; Sinus, the lesion in the renal sinus; MRI, magnetic resonance imaging; T1WI, T1-weighted images; T2WI, T2-weighted images; DWI, diffusion-weighted images; CT, computed tomography; HV, hyaline-vascular; PC, plasma-cell; MF, mixed form.

Since the treatment of CD is different from other diseases, such as renal carcinoma, lymphoma, and paraganglioma, the preoperative diagnosis of CD involving renal sinus will affect the choice of clinical treatment. UCD is mainly a hyaline vascular type, which is difficult to distinguish from other vascular-rich tumors on imaging. Most are isolated soft tissue masses with well-defined boundaries and minimal bleeding and necrosis. Satellite lesions are seen at the edge of the mass. The lesions in the corticomedullary phase are significantly enhanced, comparable to the enhancement of the aorta, so the images in this phase are easier to identify. Renal clear cell carcinoma is mostly characterized by a solid soft tissue mass with rich blood supply, which is accompanied by hemorrhage and necrosis when it is large. Most tumors show the characteristics of “fast in and fast out” on enhanced scanning, that is, the enhancement degree in the cortical phase is similar to that in the renal cortex, and the enhancement in the parenchymal phase is reduced. The above characteristics are different from UCD. Lymphoma, which includes multiple lesions, obvious enlargement of lymph nodes, fusion into masses, cystic degeneration, and necrosis can be seen, the degree of enhancement is lower than that of CD, and the surrounding blood vessels are often surrounded, that is, “vascular floating sign.” Paraganglioma: mostly solid or cystic solid mass, with bleeding and fluid level, obvious enhancement, and most of them have empty blood vessels. Cystic degeneration is rare in UCD.

However, due to the low incidence of CD in renal sinus, there is a strong likelihood of missed diagnosis or misdiagnosis, and it is, therefore, important to be aware of the risk. Heightened awareness of this disease and its radiographic manifestations may prompt consideration of this diagnosis. Therefore, it is important to be aware of the risk of this condition, and the findings from this report might prove useful to clinicians in the future. Therefore, in this case, we explored the radiologic findings to find out some radiologic features suggesting this condition to help clinicians to schedule nephron-sparing surgery in the future.

## Data Availability

The original contributions presented in the study are included in the article/Supplementary Material, further inquiries can be directed to the corresponding author/s.
